# Mass Encroaching on the Right Atrium: An Unusual Echocardiography Finding

**DOI:** 10.1002/ccr3.72540

**Published:** 2026-04-12

**Authors:** Dina El Chebabi, Nassif Feghali

**Affiliations:** ^1^ School of Medicine and Medical Sciences, Holy Spirit University of Kaslik Jounieh Lebanon; ^2^ Department of Cardiology Saint Georges Medical Center Ajaltoun Keserwan Lebanon

**Keywords:** cardiology, cardiovascular disorders, diaphragmatic eventration, echocardiography, respiratory medicine, right atrium

## Abstract

Interesting extracardiac findings can incidentally be found on transthoracic echocardiography such as a right hemidiaphragmatic eventration which can rarely be seen compressing the right atrium. Careful echocardiographic interpretation combined with clinical correlation is essential to distinguish extracardiac pseudo‐masses mimicking true intracardiac masses.

## Graphical abstract

Right hemidiaphragmatic eventration compressing the right atrium, seen on transthoracic echocardiography and confirmed by chest computed tomography, highlighting the importance of differentiating intracardiac masses from extracardiac masses when performing a cardiac echography.

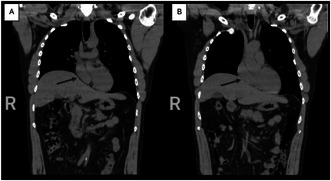

## 1 Introduction

While performing a transthoracic echocardiography, many atypical findings can be discovered, such as cardiac masses or unusual structures that potentially mimic cardiac masses. Those findings can be attributed to normal cardiac variants or remnants, pathological variants not related to specific cardiac masses, such as pericardial cysts or endocarditis, iatrogenic foreign bodies, ectopic structures, or even artifacts [1].

Abnormal findings can be intracardiac or extracardiac. The I‐mass study demonstrated that thrombus represented the majority of intracardiac masses, followed by primary benign cardiac tumors, secondary malignant tumors, then primary malignant cardiac tumors, and finally cysts [2]. While most intracardiac masses are found on the left side of the heart, rare intracardiac findings in the right atrium include angiosarcomas, lymphomas, intracardiac thrombus, and a prominent crista terminalis [3]. Concerning extracardiac findings found on transthoracic echocardiography, pleural effusion was found to be the most common, followed by ascites and liver abnormalities [4].

We present the case of a patient with a solid mass impinging on the right atrium on transthoracic echocardiography.

## 2 Case History/Examination

A 51‐year‐old male presented to the outpatient clinic complaining of dyspnea when running, even on very short distances, which appeared a few weeks back. He does not report dyspnea at rest, during minimal or everyday activity, while walking, or climbing stairs. The patient doesn't complain of orthopnea, chest pain, or any other respiratory or gastric symptoms. He maintains an active lifestyle with consistent gym attendance and participation in sports. Despite having shortness of breath on running, the patient does not complain of dyspnea when completing his strength training at the gym. The patient is a non‐smoker with a normal BMI. His medical history includes hypertension controlled on Irbesartan 150 mg/Amlodipine 5 mg once daily.

On physical examination, his vital signs were within normal limits. S1 and S2 were present with a regular rhythm, and no murmurs, rubs, or gallops were heard on cardiac auscultation. Lungs were also clear to auscultation with good air entry bilaterally. No jugular venous distention, hepatojugular reflux, or lower leg edema were noted.

## 3 Differential Diagnosis, Investigations, and Treatment

Initial assessment focused on cardiac function to rule out heart failure (with preserved or reduced ejection fraction), wall motion abnormalities, cardiomyopathies, or pulmonary hypertension.

A 12‐lead electrocardiogram (ECG) showed sinus rhythm and no abnormalities. Laboratory tests, including cardiac enzymes, were within normal reference values.

**FIGURE 1:**
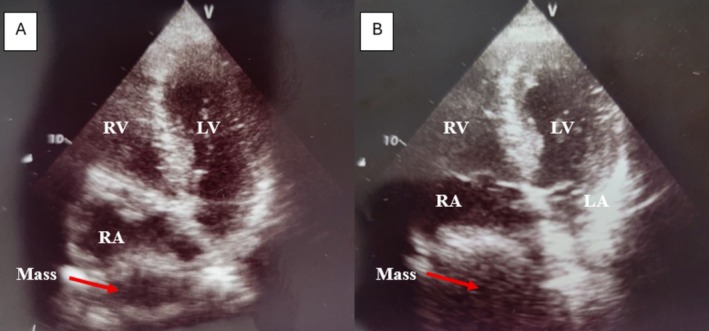
Mass encroaching on the right atrium on the four‐chamber apical view on 2‐D transthoracic echocardiography. RV = right ventricle; LV = left ventricle; RA = right atrium; LA = left atrium; Mass (Arrow).

A two‐dimensional transthoracic echocardiography was then performed. Ejection fraction was normal, no valvular abnormalities were noted, no wall motion abnormalities were depicted, and pulmonary artery pressure was also normal. The echocardiography showed an echogenic mass, probably of solid consistency, encroaching on the right atrium, especially visible on the four‐chamber apical view (Figure 1). The mass did not seem to be originating from the right atrial wall but was rather compressing it. On tissue doppler imaging, the mass had a coherent motion with the cardiac cycle. This structure did not affect the chamber's contractility or cause any hemodynamic compromise. All other echocardiography measurements were within normal limits. So, this pseudo‐mass was attributed to be of extracardiac origin.

**FIGURE 2:**
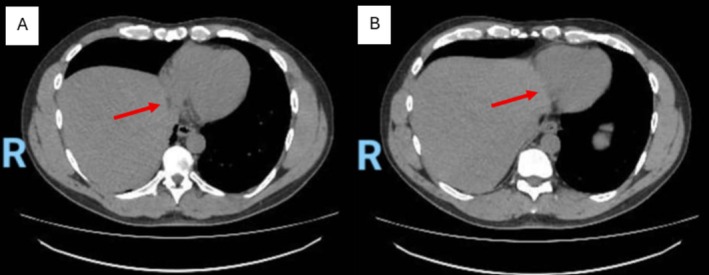
(Placeholder image for Video 1): Axial view of the thoracic CT scan, showing the liver compressing the right atrium (arrow).

**FIGURE 3:**
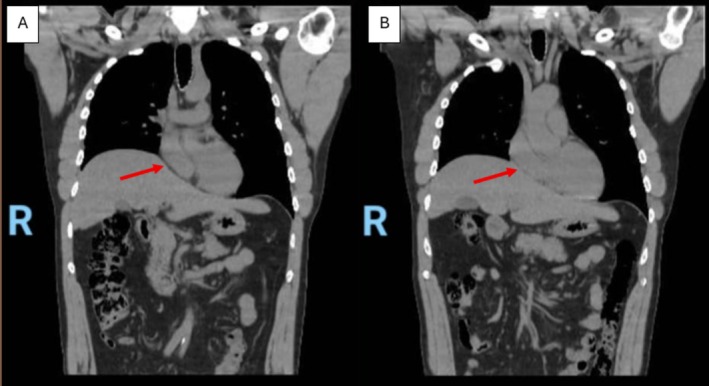
(Placeholder image for Video 2): Coronal view of the thoracic CT scan, demarcating a right hemidiaphragmatic eventration with the dome of the liver constricting the right atrium (arrow).

The patient then underwent a chest and abdominal computed tomography (CT) scan. The CT scan showed a large eventration of the right hemidiaphragm with multisegmental collapse of the basilar segments of the right lung with associated intrathoracic ascension of the liver dome. No defect was identified within the right or left hemidiaphragms. The liver was normal in size with no evidence of focal lesions (Figures 2 and 3; Videos 1 and 2).

**VIDEO 1:** Axial view of the non‐contrast thoracic CT scan, showing the liver encroaching on the right atrium. Video content can be viewed at https://onlinelibrary.wiley.com/doi/10.1002/ccr3.72540.

**VIDEO 2:** Coronal view of the non‐contrast thoracic CT scan, showing the eventration of the right hemidiaphragm, with the dome of the liver consequently compressing the right atrium. Video content can be viewed at https://onlinelibrary.wiley.com/doi/10.1002/ccr3.72540.

Since the contractility of the right atrium was normal on transthoracic echocardiography, and this mass did not cause any hemodynamic compromise, the patient's dyspnea was attributed to the right hemidiaphragmatic eventration and collapse of the basilar segments of the right lung. The patient did not have severe or restraining symptoms, so he was advised a conservative management with physical therapy.

## 4 Outcome and Follow‐Up

On follow‐up, after several sessions of physical therapy, the patient was doing well and reported a decrease in his exertional dyspnea.

## 5 Discussion

Findings on echocardiography attributed to the right atrium usually include benign cardiac tumors (Myxoma), malignant cardiac tumors (Angiosarcomas), secondary cardiac involvement by lymphoma or metastases, or noncancerous findings such as a thrombus or embryologic remnants such as a prominent crista terminalis [3]. While extracardiac findings can depict liver abnormalities, those found on transthoracic echocardiography usually include liver cysts, gallstones, and less commonly malignant liver masses [4].

It is usually difficult to differentiate between a true intracardiac right atrium mass and a pseudo‐mass on TTE, although some echocardiography features can help. Specifically, color tissue doppler imaging (TDI) and pulsed‐wave tissue doppler imaging (PW‐TDI) may help with the distinction of intracardiac pathological structures through different color coding and motion compared to the surrounding cardiac tissue. A tissue doppler imaging characterized by incoherent motion of the right atrial mass compared to the cardiac motion is typical of right‐sided thrombi, myxoma, or vegetation, whereas a synchronous and concordant motion with the cardiac cycle refers to pseudo‐masses [5, 6].

In our case, diagnoses of right atrial thrombus and myxoma were eliminated since the pseudo‐mass' motion was coherent with the cardiac cycle on TDI, and the mass was not seen originating inside the right atrium, but it was rather compressing from outside [6]. A prominent crista terminalis was eliminated as well since the echogenicity of the pseudo‐mass was different than that of the atrial wall, unlike a prominent crista terminalis that would present with an echogenicity similar to that of the adjacent myocardium [7]. A pericardial cyst diagnosis was also excluded since the pseudo‐mass in our case seemed to have a solid consistency, while a pericardial cyst is filled with fluid and appears anechoic on TTE [8].

Although our patient presented with dyspnea, which is the most common presentation of a patient with cardiac angiosarcoma, the patient did not present any other symptoms, and the echography features were not suggestive of a malignant process. The mass did not have an attachment to the endocardial surface and was not obstructive of the tricuspid valve or right ventricular outflow tract. There was no pericardial effusion or tamponade [9]. Following these findings, a cardiac angiosarcoma was eliminated.

Since the mass in our case was most likely of solid consistency, not originating from the right atrium, but rather compressing it, with no malignant features, a chest CT scan was ordered to diagnose the extracardiac finding that was shown on echocardiography compressing the heart. The chest CT then revealed a right hemidiaphragmatic eventration.

Hemidiaphragmatic eventration refers to the elevation of one side of the diaphragm without any rupture in the continuity of the diaphragm. Patients are usually asymptomatic, and the condition is usually discovered on X‐ray incidentally. When symptomatic, patients may present with respiratory or gastrointestinal symptoms [10]. The most common presentation is dyspnea due to impaired ventilation. In hemidiaphragmatic eventration, caudal movement of the diaphragm is reduced, and blood flow to the basal region of the lung on the same side as the affected diaphragm may also be impaired, possibly due to regional vasoconstriction triggered by alveolar hypoxia. Together, these will result in a ventilation/perfusion mismatch and decreased chest wall compliance, leading to dyspnea [11].

Patients are usually treated with conservative management, including physical therapy and pulmonary rehabilitation [10]. Patients who experience failure of medical management or those with severe cases requiring mechanical ventilation are candidates for surgical plication [11].

Complete eventration of the hemidiaphragm is usually seen on the left side, and complete eventration of the right hemidiaphragm is rare but has been reported in the literature [12].

What is even more rare is to be able to find the dome of the liver compressing the right atrium on 2‐D echocardiography. This rare finding has been reported once previously, by Lau G et al. [13] who described the case of a 77‐year‐old woman presenting for subacute dyspnea. The patient had elevated troponin and was managed for acute coronary syndrome. On echocardiography, she had right coronary artery territory hypokinesis with only mild disease on cardiac angiography, and she also exhibited a mass extrinsically compressing the right atrium that was diagnosed as a right hemidiaphragmatic eventration on chest CT. In our case, the patient had normal troponin and normal echographic features apart from the pseudo‐mass impinging on the right atrium. This is the second case of a right hemidiaphragmatic hernia discovered on echocardiography in the literature and highlights the importance of differentiating intracardiac masses from extracardiac masses on TTE to later guide the selection of proper supplementary imaging and establish an accurate diagnosis.

## 6 Conclusion

This case highlights the rare presentation of a right hemidiaphragmatic eventration, seen compressing the right atrium on 2‐D transthoracic echocardiography. It emphasizes the importance of exhibiting meticulous attention to all chambers when performing an echocardiography, as extracardiac pathologies can sometimes be diagnosed.

## Author Contributions

**Dina El Chebabi:** conceptualization, data curation, investigation, resources, writing – original draft. **Nassif Feghali:** conceptualization, supervision, writing – review and editing.

## Funding

The authors have nothing to report.

## Consent

The authors certify that they have obtained all appropriate patient consent forms. The patient has given his written informed consent for publishing the case report.

## Conflicts of Interest

The authors declare no conflicts of interest.

## Data Availability

All relevant anonymized data supporting this case report are included within the article and its Supporting Information.
